# Staging Dementia From Symptom Profiles on a Care Partner Website

**DOI:** 10.2196/jmir.2461

**Published:** 2013-08-07

**Authors:** Kenneth Rockwood, Matthew Richard, Chris Leibman, Lisa Mucha, Arnold Mitnitski

**Affiliations:** ^1^Dalhousie UniversityDepartment of MedicineDalhousie UniversityHalifax, NSCanada; ^2^DGI Clinical IncHalifax, NSCanada; ^3^Janssen Alzheimer Immunotherapy Research and Development, LLCSouth San Francisco, CAUnited States; ^4^Pfizer IncCollegeville, PAUnited States

**Keywords:** dementia, staging, online survey, symptoms, Cognitive Impairment Not Dementia, Mild Cognitive Impairment, validation, Artificial Neural Networks, World Wide Web

## Abstract

**Background:**

The World Wide Web allows access to patient/care partner perspectives on the lived experience of dementia. We were interested in how symptoms that care partners target for tracking relate to dementia stage, and whether dementia could be staged using only these online profiles of targeted symptoms.

**Objectives:**

To use clinical data where the dementia stage is known to develop a model that classifies an individual’s stage of dementia based on their symptom profile and to apply this model to classify dementia stages for subjects from a Web-based dataset.

**Methods:**

An Artificial Neural Network (ANN) was used to identify the relationships between the dementia stages and individualized profiles of people with dementia obtained from the 60-item SymptomGuide (SG). The clinic-based training dataset (n=320), with known dementia stages, was used to create an ANN model for classifying stages in Web-based users (n=1930).

**Results:**

The ANN model was trained in 66% of the 320 Memory Clinic patients, with the remaining 34% used to test its accuracy in classification. Training and testing staging distributions were not significantly different. In the 1930 Web-based profiles, 309 people (16%) were classified as having mild cognitive impairment, 36% as mild dementia, 29% as moderate, and 19% as severe. In both the clinical and Web-based symptom profiles, most symptoms became more common as the stage of dementia worsened (eg, mean 5.6 SD 5.9 symptoms in the MCI group versus 11.9 SD 11.3 in the severe). Overall, Web profiles recorded more symptoms (mean 7.1 SD 8.0) than did clinic ones (mean 5.5 SD 1.8). Even so, symptom profiles were relatively similar between the Web-based and clinical datasets.

**Conclusion:**

Symptoms targeted for online tracking by care partners of people with dementia can be used to stage dementia. Even so, caution is needed to assure the validity of data collected online as the current staging algorithm should be seen as an initial step.

## Introduction

The World Wide Web offers new opportunities for understanding disease from a patient’s standpoint, and crucially in dementia, from the standpoint of their caregivers [1]. On the Web, detailed information can be collected from survey data [2,3] or extracted from online programs offered to caregivers [4-8]. In any examination of patient/caregiver perspectives on the lived experience of dementia, understanding the stage of dementia being discussed is crucial. Unfortunately, how best to stage dementia using caregiver reports on the Internet is not clear. Earlier, we have shown that a structured questionnaire based on the Dependence Scale [9] designed to grade increasing degrees of dependence showed good construct validity as a staging measure [2]. While structured questionnaires can be employed, users can see them as intrusive and unrewarding, especially if their completion seems to require undue effort. Even so, it is very important for caregivers to have some sense about dementia stage since many disease manifestations are stage dependent (eg, wandering is a later stage symptom); whereas in other cases, a symptom appearing “out of order” (in relation to untreated Alzheimer’s disease) would have diagnostic value (eg, hallucinations occurring very early in the dementia course would suggest Lewy Body disease).

The SymptomGuide for dementia is a Web-based tool aimed particularly at the caregivers of persons with dementia. This tool allows a caregiver to track the health status and symptoms of the person they are giving care to. In addition, using its corresponding online symptom library, caregivers can learn more about common manifestations of dementia [10]. We were interested in using this information as a means of staging dementia. Of the many instruments commonly used clinically to stage dementia, none relies only on symptoms. Constructing algorithms to stage dementia from symptoms alone is fundamentally challenging using a priori rules since many common symptoms can occur at different stages of dementia. In general, the complex relationships between clearly associated symptoms and dementia stages are difficult to discern using conventional classical statistical methods. By contrast, artificial intelligence systems can approximate the complex nonlinear relationships between these variables including outcomes [11]. Artificial Neural Network (ANN) machine-learning techniques could be particularly beneficial in discovering patterns and how they change with disease progression. ANNs have been applied in the discrimination of mild cognitive impairment (MCI) from Alzheimer’s disease [12] and to identify risk factors on the conversion of amnestic mild cognitive impairment [13], as well as in analysis of neuroimaging data [14,15]. ANNs have also been applied to associating individual characteristics with outcomes [16]. ANNs have been compared with conventional statistical approaches [17] and in particular in Alzheimer’s disease research [18], suggesting the usefulness of this approach.

Our overall objective was to develop an application of a machine-learning ANN algorithm to stage dementia using Web-based, individualized symptom profiles. In particular, we aimed to (1) develop and validate a symptom-based staging system using memory clinic data, where staging can be verified, (2) apply this to the Web-based symptom data, and (3) explore differences in online and memory clinic symptom targeting that might influence staging and its interpretation.

## Methods

### Setting

The data came from the SymptomGuide (SG) website (see Multimedia Appendix 1). Caregivers can visit this site to learn about symptoms exhibited by the person they are caring for. Individuals with dementia can also input data; although, only 1% of this site’s users report having dementia themselves. Crucially for these analyses, caregivers target the symptoms most relevant to them in order to track the course of the disease and/or the effects of treatment [11] (people being profiled on the Web may have many more symptoms than those being targeted for tracking; these profiles need to be interpreted as statements about the most troubling symptoms and not as symptom inventories). The online symptom library (Multimedia Appendix 2) describes 60 symptoms, each detailed using about a dozen plain-language descriptors [19]. The library defines and describes each symptom and includes information about the typical stage of dementia when that symptom occurs. Subcategories in the library provide other relevant information, accessed by clicking on tabs visible for each symptom (eg, a tab entitled “Doctor’s Diary” provides standard advice from a physician about the typical challenges and course related to that problem). One heavily trafficked subsection describes common management strategies that can be employed in relation to each symptom. For users who build symptom profiles, learning about and tracking symptoms is their chief interaction with the site, so that building a staging algorithm from the patient profiles does not require additional effort by care partners.

Between its launch in 2007 and March 2012, 6129 online users have built symptom profiles, of whom 1930 have also created complete personal profiles, which consist of data about demographics, medications, symptoms, and symptom progression. These data were considered the Web-based data for this study.

### Measures

In addition to Web-based users, the SG is used in a tertiary care Memory Clinic in Halifax, Nova Scotia. Each of the 323 clinic-based SG users whose data are considered here underwent standard assessments, which included staging based on the Global Deterioration Scale (GDS) score for dementia [20] (determined during the clinical interview by the examining physician as the mean value of the first 5 axes of the Brief Cognitive Rating Scale [21]). The GDS was scored as 3=cognitive impairment not dementia (CIND)/mild cognitive impairment, 4=mild dementia, 5=moderate dementia, and 6=severe dementia. A Mini-Mental State Examination (MMSE, usually administered by a clinic nurse) was also scored. The MMSE is a screening cognitive test, scored from 0-30, with a higher score indicating better performance. The GDS rater was an experienced clinician scientist (the first author, KR) who was not blind to the MMSE. Earlier work had suggested that a detailed questionnaire for staging was not of interest to most users. Here we substituted a brief questionnaire that asked if the person being profiled has been diagnosed with dementia and then described each stage in a single sentence. Users were asked which sentence best described the person being profiled.

### Analysis

A symptom-derived staging algorithm is proposed to recognize four major groupings of cognitive impairment: MCI, mild, moderate, and severe dementia. The algorithm was developed (Objective 1) from profiles in the Memory Clinic (where staging is known) and applied to the Web dataset (where staging usually is not contained in the database).

To develop the algorithm, we used an ANN, which consists of processing units that are called “neurons” because of certain similarities with human neurons that respond to input stimuli in a nonlinear fashion. The algorithm recursively analyzes their ability to predict an individual’s dementia stage, given the information about that individual’s symptoms and staging. The ANN was applied to a random selection of 66% (211/320) of the Memory Clinic database profiles to train the ANN. The remaining 34% of the clinic sample was used to assess the accuracy of the ANN model. The input variables for the ANN model were the presence or absence of SG symptoms and the person’s stage. Of the 60 symptoms presented in the SG, we used the 34 that had been used in at least 5% of the Web dataset and at least 5 times in the clinical dataset. Parameters of the ANN model included 4 nodes, an over fit penalty of 0.15, a 0.00001 convergence criteria, and 5 tours of 500 iterations. The output variables were the predicted probabilities of the 4 dementia stages. These specific ANN model parameters were chosen to optimize the percent of correctly predicted stages in the test data (ie, the 34% of clinic records not used in the training set). To test the robustness of the model, each training session was repeated 30 times; stability was tested using the coefficient of variation, with a tolerance of 15% change in classification.

The ANN model found using the clinic data was cross-validated in that dataset by correlating it with the MMSE and presentation, box-plot diagrams (Objective 1). It was then used to predict the stage of dementia in the Web-based sample (N=1930), for whom no stage of dementia was known (Objective 2). To explore how symptom targeting online might differ from symptom targeting in the Memory Clinic (Objective 3), we first cross-tabulated symptom profiles by stage of dementia for both the known clinical dataset and the predicted Web-based dataset. Next, we compared the number of symptoms set in each sample, again by dementia stage. We also explored how commonly Analysis of Variance (Kruskal-Wallis ANOVA) was used to measure group mean differences in MMSE by stage. The Pearson chi-square test was used to test for differences between staging distributions, using a significance level of 0.01. Calculations and analysis were performed using R statistical software v2.14.2.

### Ethics

The study was approved by the Research Ethics Committee of the Capital District Health Authority, Nova Scotia. Clinic participants signed informed consent. All respondents to the survey consented by checking their agreement to a terms and conditions list, which included their consent to the use of anonymized data. No personal information was collected that could identify the survey participant. All responses are stored on a secure server.

## Results

### Memory Clinic

The Memory Clinic patients were of a similar age to Web users, but more of the latter were women, and fewer Web users lived with family members (Table 1).

**Figure 1:**
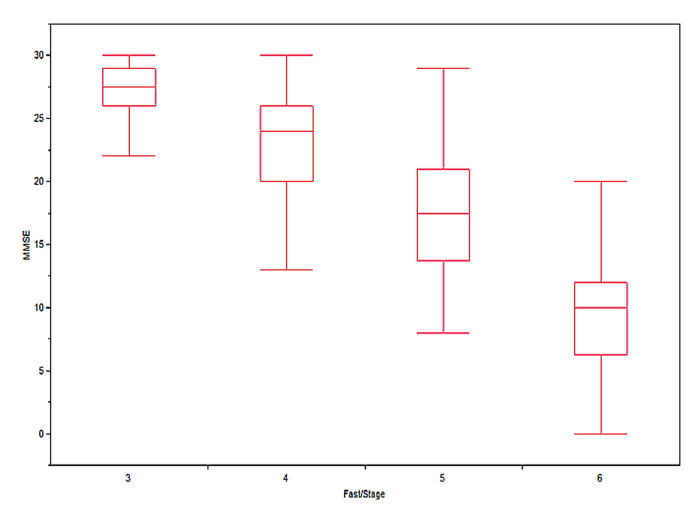
The association between Mini-Mental State Examination scores and dementia stages in Memory Clinic patients (N=109) staged according to ANN staging algorithm.

The machine-learning algorithm developed in the Memory Clinic training dataset showed virtually the same dementia stage distribution when applied to the testing dataset as did the clinical dementia staging. By both staging assignments, most patients had mild dementia (55% and 61%, respectively), followed by CIND/MCI (23% and 20%), moderate dementia (14%, 12%), and severe dementia (8%, 7%). Each training session was repeated 30 times; the coefficient of variation never exceeded 12% for each stage. The final ANN model showed a misclassification rate of 3%. In the Memory Clinic dataset, the ANN staging algorithm was significantly related to the MMSE scores (*F*_3,26_=101.1, *P*<.001) (Figure 1).

**Figure 2:**
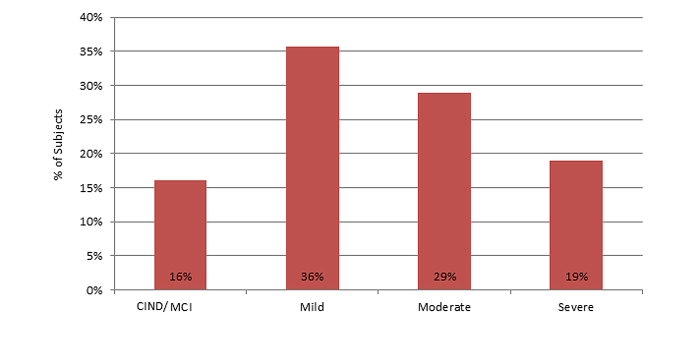
Distribution of Web-based users by clinical stages as classified by the ANN model staging algorithm (n=1930).

In the Web-based dataset, most people were in the mild stage (Figure 2).

### Comparison of Symptom Profiling Between the Memory Clinic and the Online Datasets

**Figure 3:**
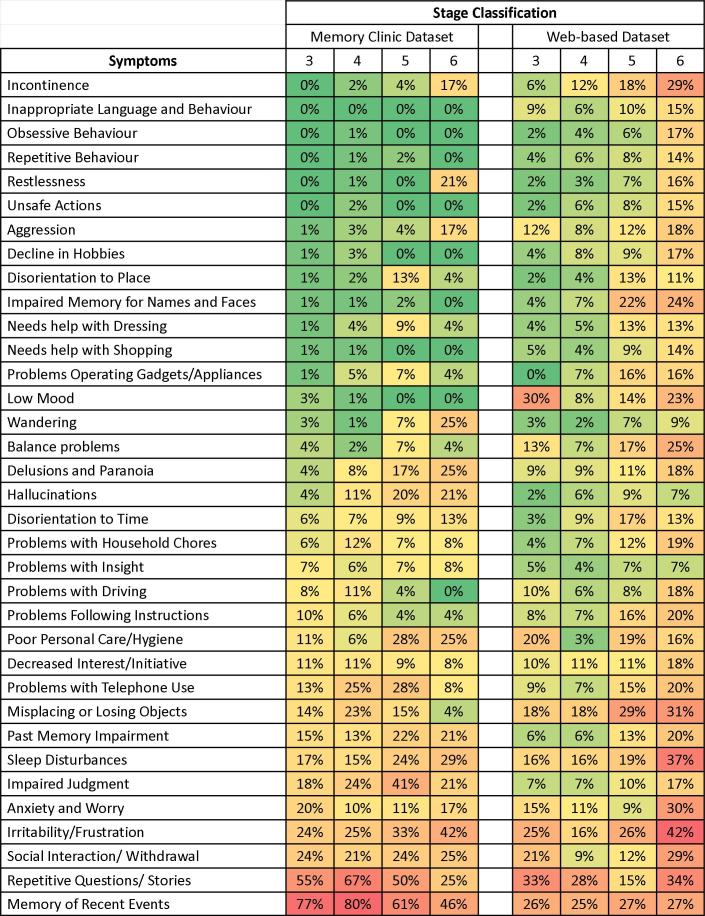
Comparison of the distribution of symptoms between the Memory Clinic and Web-users. Colour represents symptom frequencies: green (lower frequencies) to yellow (intermediate) to red (higher frequencies).

Symptom profiles were relatively similar between the Web-based and clinical datasets (Figure 3). Three trends were evident. First, even the most common symptoms selected for tracking in the Web-based dataset occur in less than half the profiles. This is also true for all but two symptoms in the Memory Clinic dataset. Even so, in general, the symptom profile of the Web-based dataset showed slightly higher symptom occurrence rates when compared to the clinical dataset. Overall, people who used the website targeted more symptoms (mean 7.1 SD 8.0) than did people in the Memory Clinic (5.5 SD 1.8) (*t*_2101_=-7.69, [Welch’s *t* test for unequal sample sizes and unequal variances], *P*<.001). This appears to arise as a consequence of the third trend, which is that symptom targeting rates increased as the dementia severity stage progressed into stages 5 and 6. Specifically, Web users whose profiles conformed to stage 3 had a mean 5.6±4.9 symptoms vs people in Memory Clinic, (4.97 SD 1.94, *t*_285_=-1.73, *P*=.083); those in stage 4 targeted 4.8 SD 5.6 symptoms vs clinical mean 5.7 SD 1.61 (*t*_854_=3.69, *P*<.001); in stage 5, 7.8 SD 7.9 symptoms vs clinical mean 6.07 SD 2.22 (*t*_173_=-3.71, *P*<.001); and in stage 6, 11.9 SD 11.3 vs clinical mean 5.67 SD 1.97 (*t*_17_=-8.72, *P*<.001). Given that the Web-based dataset had more patients in the moderate (29%) and the severe (19%) stages than did the Memory Clinic dataset (14% moderate and 8% severe), this appears to account for the difference in the mean number of symptoms between the two groups (chi-square_3_=71.3, *P*<.001).

**Table 1:** Demographic characteristics of Memory Clinic patients and Web users.

Demographics		Memory clinic data N=320	Web-based data N=1930
Mean age, years (SD)		72 (10.4)	74 (10.4)
% female		46	59
**Living arrangements, %**			
	Alone	7	20
	With spouse/family or friend	90	58
	Care facility/ nursing home	3	22

## Discussion

### Principal Findings

This paper used a machine-learning, symptom-based staging algorithm, developed from a Memory Clinic database of symptoms and stages, to define stages using only symptoms in a Web-based dataset. The Web-based dataset recorded symptoms targeted for treatment by care providers of people with dementia (and in a very few cases, by people with dementia themselves). Using an ANN model with 34 symptoms as inputs, consistent dementia stage classification was possible. The staging algorithm was trained and its accuracy tested using clinical data, where the stage of dementia for each patient was established according to the standard diagnostic. The misclassification rate in the Memory Clinic data was low (3%) and indicated a very good performance of the ANN model when validated internally (ie, using the Memory Clinic data). It was not possible to validate the model externally (in the Web-based data) in the same way as was done in the Memory Clinic because different criteria were used in both datasets to define the stages. In the Memory Clinic, staging was done by clinicians using the judgment-based GDS.

ANN models are known to be powerful machine-learning techniques which, when properly trained and tested, can give reliable predictions for unknown variables of interest. Until comparatively recently, their applicability in medical research was fairly limited due to the restriction of computer processing power and the lack of special training. Recent versions of major statistical packages now allow for user-friendly ANN analysis options (eg, JMP, MATLAB, Weka, R). Another major drawback in the application of ANNs is the difficulty in interpreting the results. This is because complex nonlinear relationships do not yield simple interpretations of the relationships between input variables (eg, symptoms) and response variables in a cause-effect manner. While such “black box*”* techniques show abundant applications in engineering, technical physics, and computer science, they are often less favorably received in the biomedical community, as they do not provide insight into relationships among variables. Even so, despite the desirable goal of understanding relationships among variables, the high dimensionality of problems like dementia (the dimensionality here is represented by many symptoms) makes such links nearly impossible. In situations like this, the application of ANNs presents a viable alternative for bypassing the immense complexity issue of our data and creating a model that, even though the relationship is unknown, can still reliably predict response variables by properly training and testing subsets of our data. Of note here, the simplest training (based only on associations within the symptom profile) was equally as informative as more complex algorithms, such as ones employing symptom severity, domain aggregation, and ratio of domain frequencies (eg, ratio of symptoms in the functional domain to symptoms in the behavioral domain).

The usual contrast to “bioinformatic” techniques such as ANNs, is to use “biostatistical” ones, such as factor analysis or its variant. The latter approaches, however, can sometimes ignore items that can be highly informative for individuals but are not “statistically significant” at the group level. The additive value of effects which themselves can be negligible was recently illustrated in dementia epidemiology, in which a risk factor index made up of items that did not significantly predict dementia individually was more powerful than any single traditional risk factor in dementia prediction [22]. In short, in situations of high dimensionality, tradeoffs will be needed according to analytical intent. Here, the intent is to include the patient/caregiver perspective, using as much information as possible.

Our data should be interpreted with caution. The results reflect experience in using this emerging technology (in developing a model) rather than a claim to have developed a perfectly valid and accurate model. In short, the current model should be seen as an initial step. More specifically, despite similar symptom patterns in the Web-based data and the more controlled clinical environment data, one does not map exactly to the other. On the other hand, these differences may possibly reflect actual dementia severity differences between Web-based users and clinic patients. More studies should be done in order to better understand if this is the case. Likewise, for model stability, we used only symptoms that had been used at least 5 times in the training dataset, resulting in 26 of the SG’s 60 symptoms not being used in the staging algorithm. When the training dataset includes >1000 people, we plan to reassess the algorithm to evaluate its stability and the impact of less common symptoms on staging.

Further changes to the website now allow users to make their own staging assessment based on functional, behavioral, and cognitive symptom profiles, and this too will be re-evaluated periodically. In consequence, the current staging algorithm should be seen as an initial step. Even so, the initial results suggest caution in brief summary staging measures. In contrast to the more detailed Dependence Scale, which was cumbersome for many users, a very brief staging method did not improve uptake and made precision worse. A brief staging questionnaire, which described each stage in a single sentence, was completed by only 207 people, with weak (*r*=0.32) Spearman correlation with the ANN staging. Overall, the algorithm classified 37% of people into their observer-assessed category; this improved to 83% being classified within one level. Misclassification was normally distributed about 0. On the other hand, this lack of agreement may itself be informative. While it is the case that descriptions that are standard but brief enough to be completed by users may lack validity, the discrepancy between the staging algorithm and the brief questionnaire might in fact reflect the effect of treatment. The clinic-based profiles were weighted to patients prior to treatment (ie, at the time of initial diagnosis) but include many people who have been on treatment for months to years who are being reassessed. This would also be true of Web-based users. Given that currently used medications can alleviate some symptoms but do not cure or even halt progression, the stages detected by that algorithm might correspond less well to staging based on the untreated natural history, as was the case with the brief questionnaire. In addition, in each case we are mainly looking at the caregiver’s impression of the symptoms, especially if the dementia is more than mild. Very few profiles (<1%) appear to be completed by the patient alone, and these are mostly weighted to those with MCI. The extent to which patients’ insights into their own deficits might invalidate their own accounts (or even influence their caregivers) is not known.

Even so, it is inherent in a Web database that less strict quality control is possible compared to a clinical database. In consequence, the information needs to be interpreted with caution. However, because the Web database allows a less medicalized interaction for users, it may offer additional insights on the lived experience of dementia. Clinic-based datasets rarely assay symptoms beyond what exists in standardized scales and checklists, which typically do not include the same depth of information as here and typically record information on fewer people. This is an inherent trade-off, but the current experience suggests that the Web has great potential to provide useful information. In this regard, we were struck that, taking into account stage differences, the patterns were generally similar between clinics, where symptom choice is more influenced by interactions with health care professionals and completion of standard questionnaires, and online, where it appears that most people are doing this at home without such prompting.

Being able to stage dementia using Web profiles is useful in lessening the response burden of users. It also allows naturally occurring profiles to be used, enhancing the user’s sense of contribution, instead of just completing questionnaires. More importantly, as more people with dementia are now being treated, many of the traditional staging algorithms need to be revisited. No current treatment is curative, so different combinations of mild, moderate, and severe staging items are seen, especially in patients who have been on treatment for more than a year or two. As the database grows, it should be possible to explore these relationships better. Of note, the clinical dataset that trained the algorithm included symptoms for people both receiving and not receiving treatment, so it reflects this new reality.

We found it interesting that, compared with the Memory Clinic database, in the Web-based data, symptom targeting rates increased as the dementia severity stage increased. This may reflect that Web-based users have more severe problems compared to Memory Clinic patients. Alternately, the Memory Clinic patients may have these problems too, but they are not being targeted; this is a proposition that needs to be tested.

### Conclusions

In general, robust classification of such a large sample of Web-based users allows for additional studies to be performed that reflect this perspective, including people who do not have access to memory clinic services. If further validated, it can provide a self-assessing staging classification that a caregiver can perform without additional training. Even so, lack of a means of verifying information is one reason that online data must be treated with caution. Finally, especially as disease-modifying drugs are developed that modify the course of dementia (and thereby its stages), it could lead to the creation of a more robust clinical staging methodology that considers symptom profile composition as important to understanding dementia severity and potential treatment effects. These considerations are motivating additional inquiries by our group.

## Multimedia Appendix 1

SymptomGuide screenshots.

## Multimedia Appendix 2

The online Symptom Library.

## Abbreviations

ANOVA: analysis of variance
ANN: artificial neural network
CIND: cognitive impairment no dementia
MCI: mild cognitive impairment
MMSE: Mini-Mental State Examination
