# Supplementary material for: Staging Dementia From Symptom Profiles on a Care Partner Website
Source: J Med Internet Res. 2013 Aug 7;15(8):e145. doi: 10.2196/jmir.2461 (PMC3742393; doi:10.2196/jmir.2461)
Supplement: Supplementary file 1 [file jmir_v15i8e145_app1.pptx]

## Slide 1
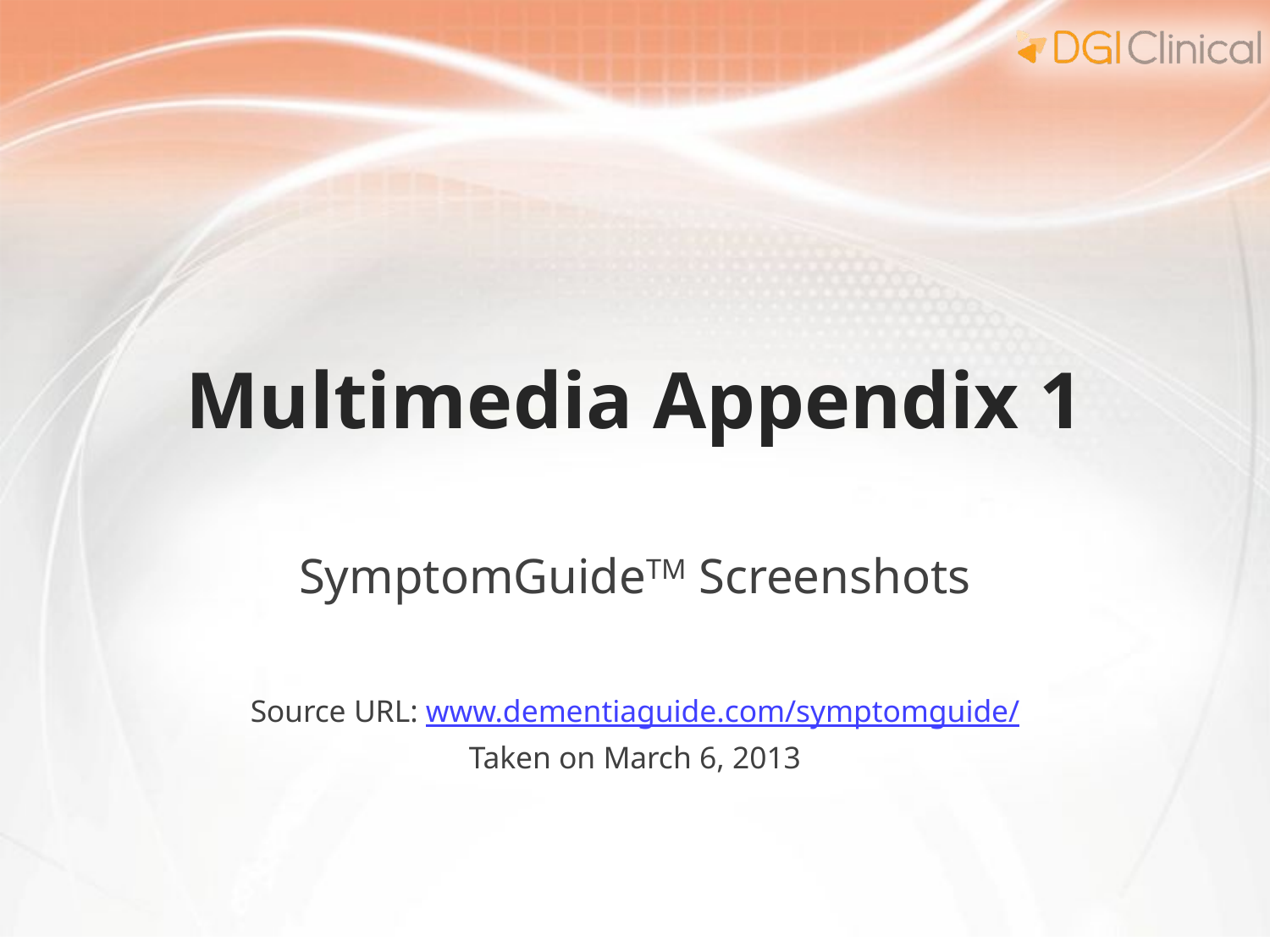

# Multimedia Appendix 1
SymptomGuideTM Screenshots
Source URL: www.dementiaguide.com/symptomguide/
Taken on March 6, 2013

## Slide 2
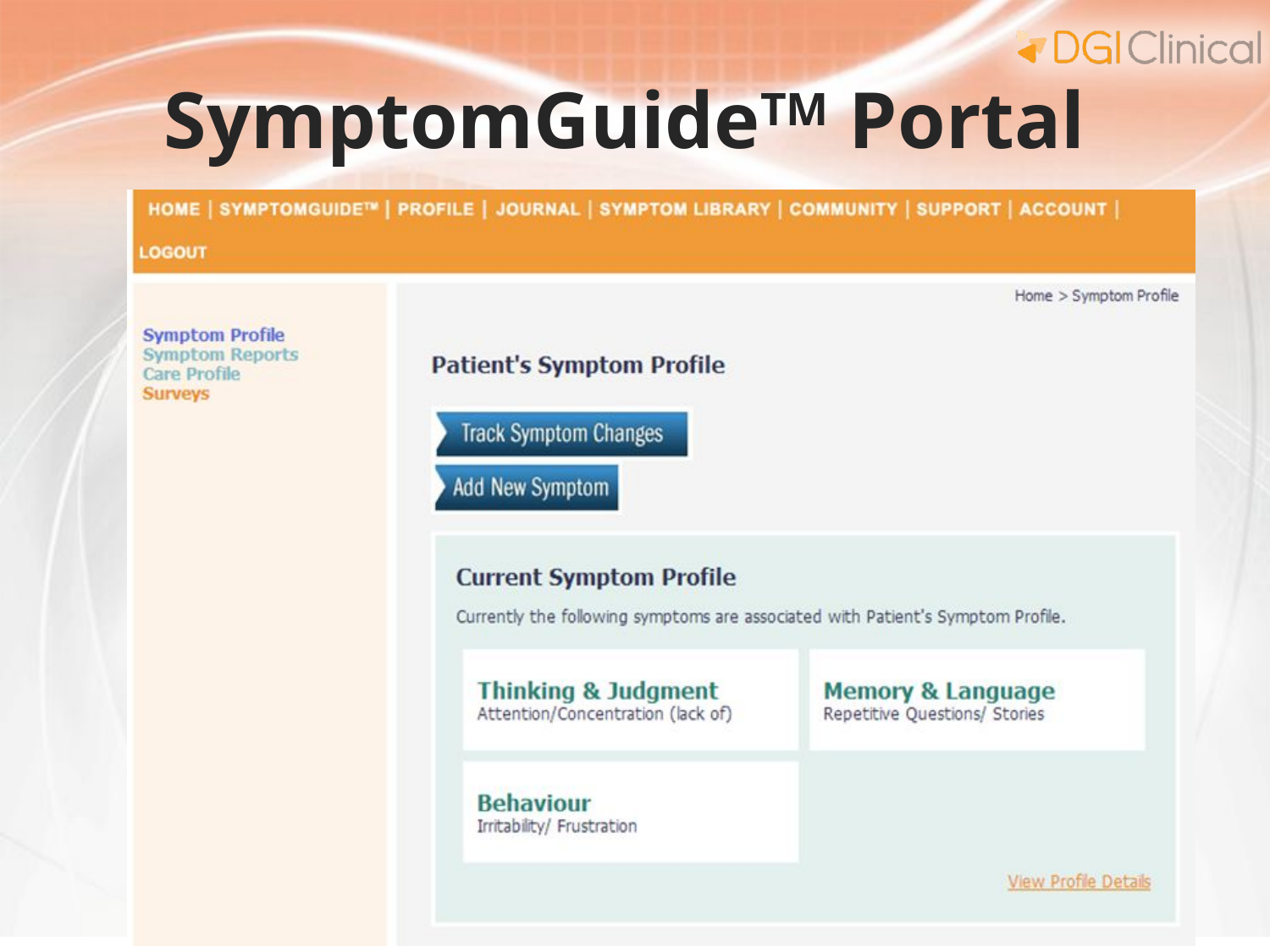

# SymptomGuideTM Portal

## Slide 3
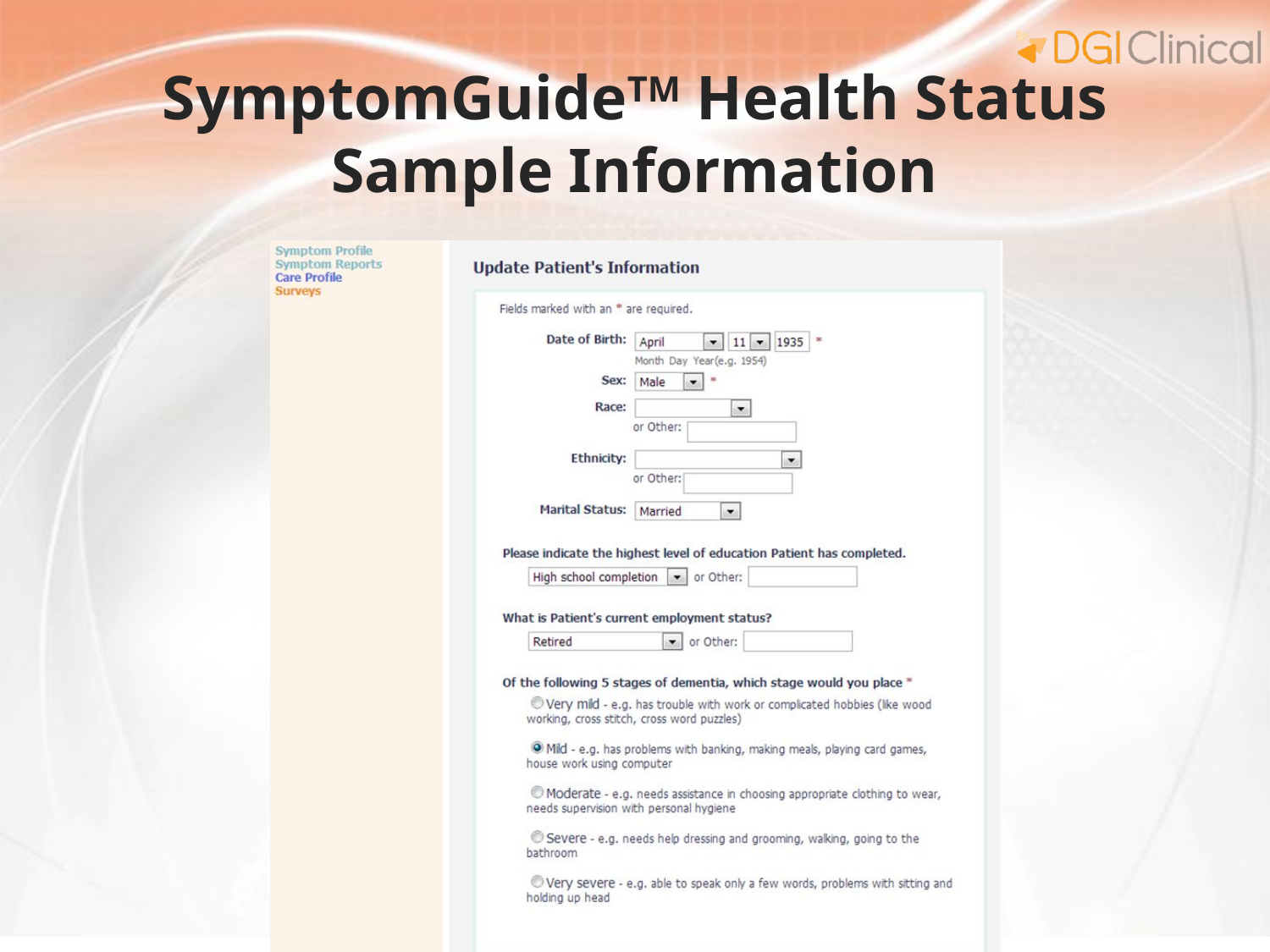

# SymptomGuideTM Health Status Sample Information

## Slide 4
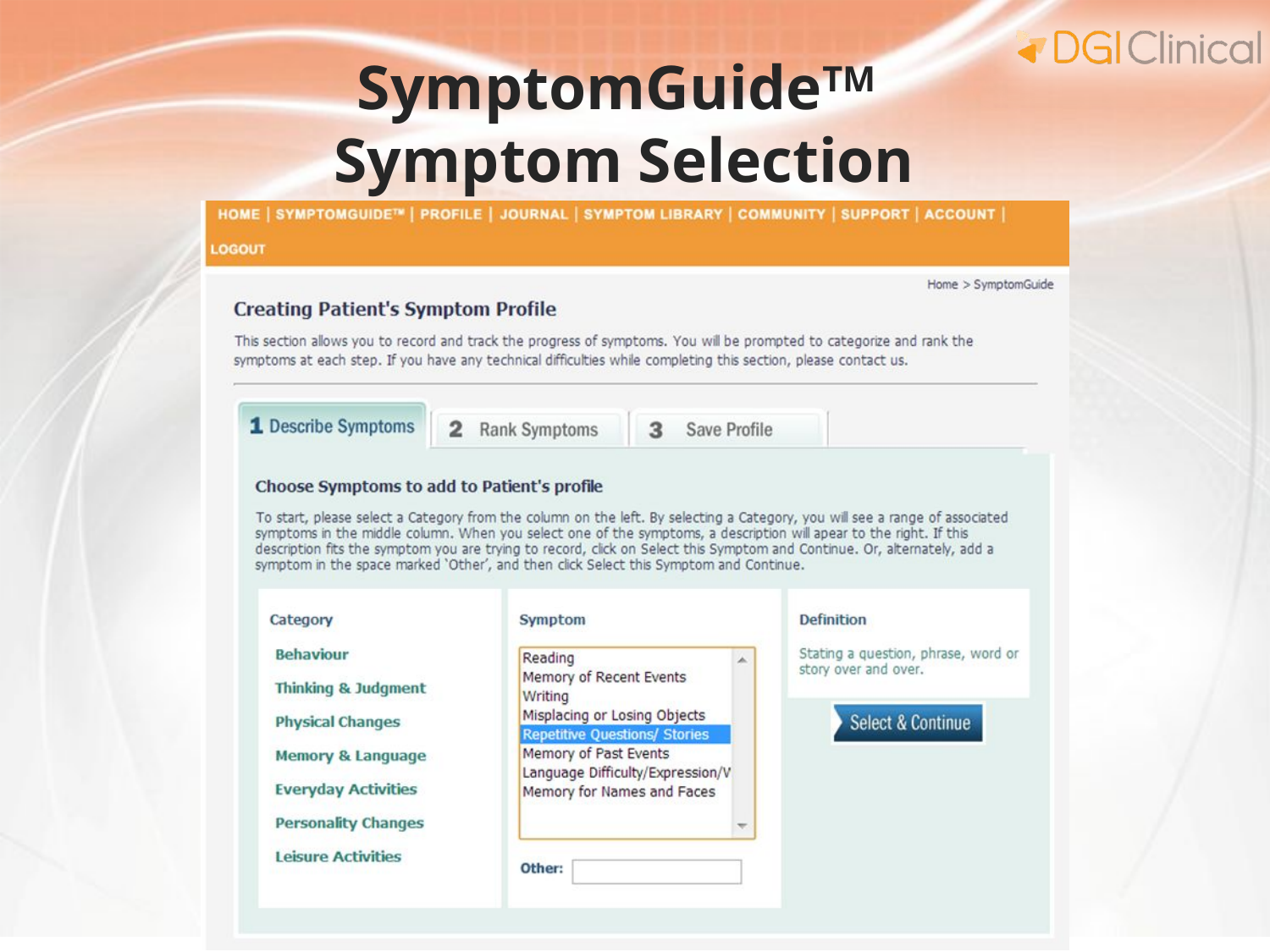

# SymptomGuideTM Symptom Selection

## Slide 5
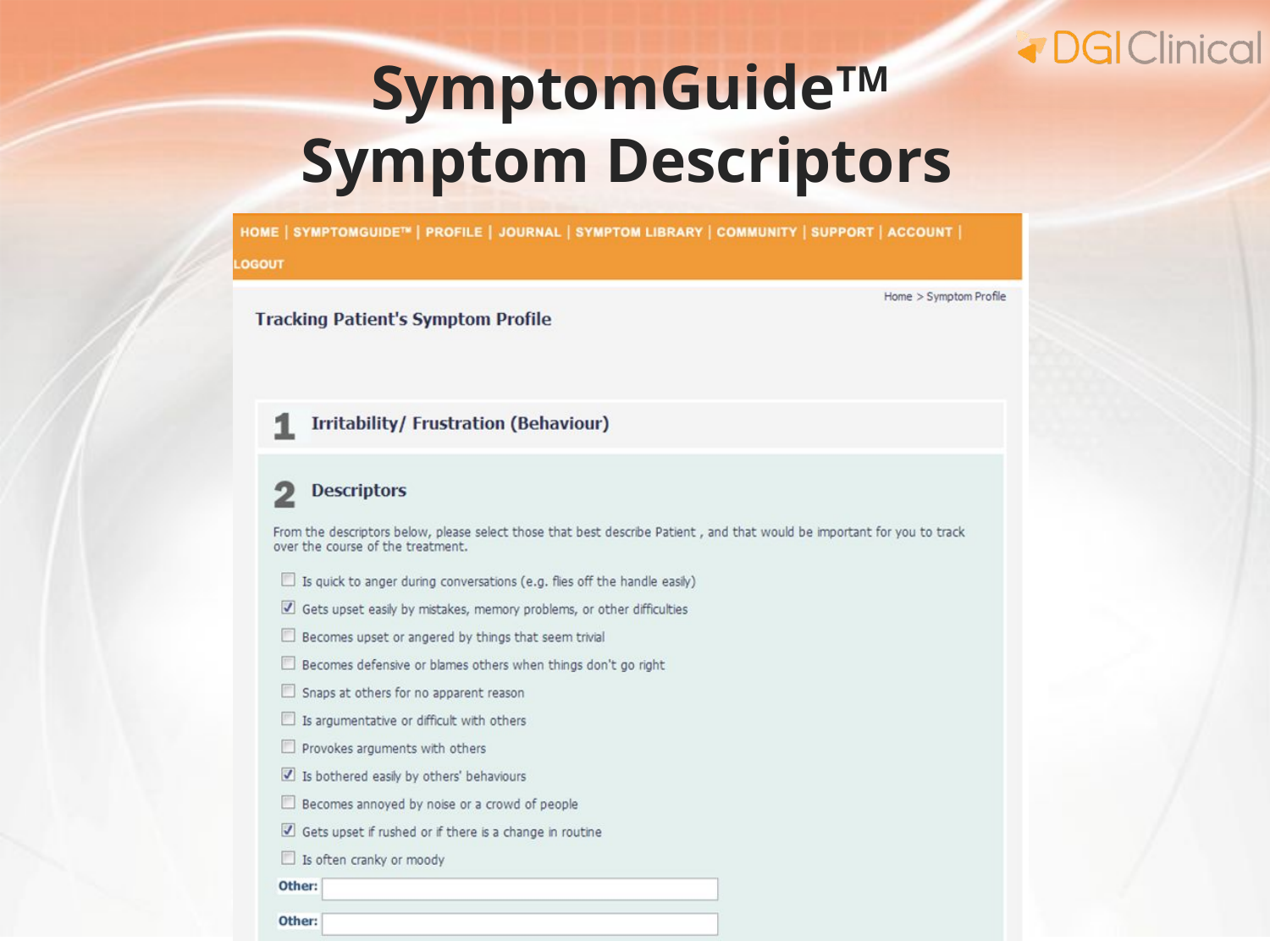

# SymptomGuideTM Symptom Descriptors

## Slide 6
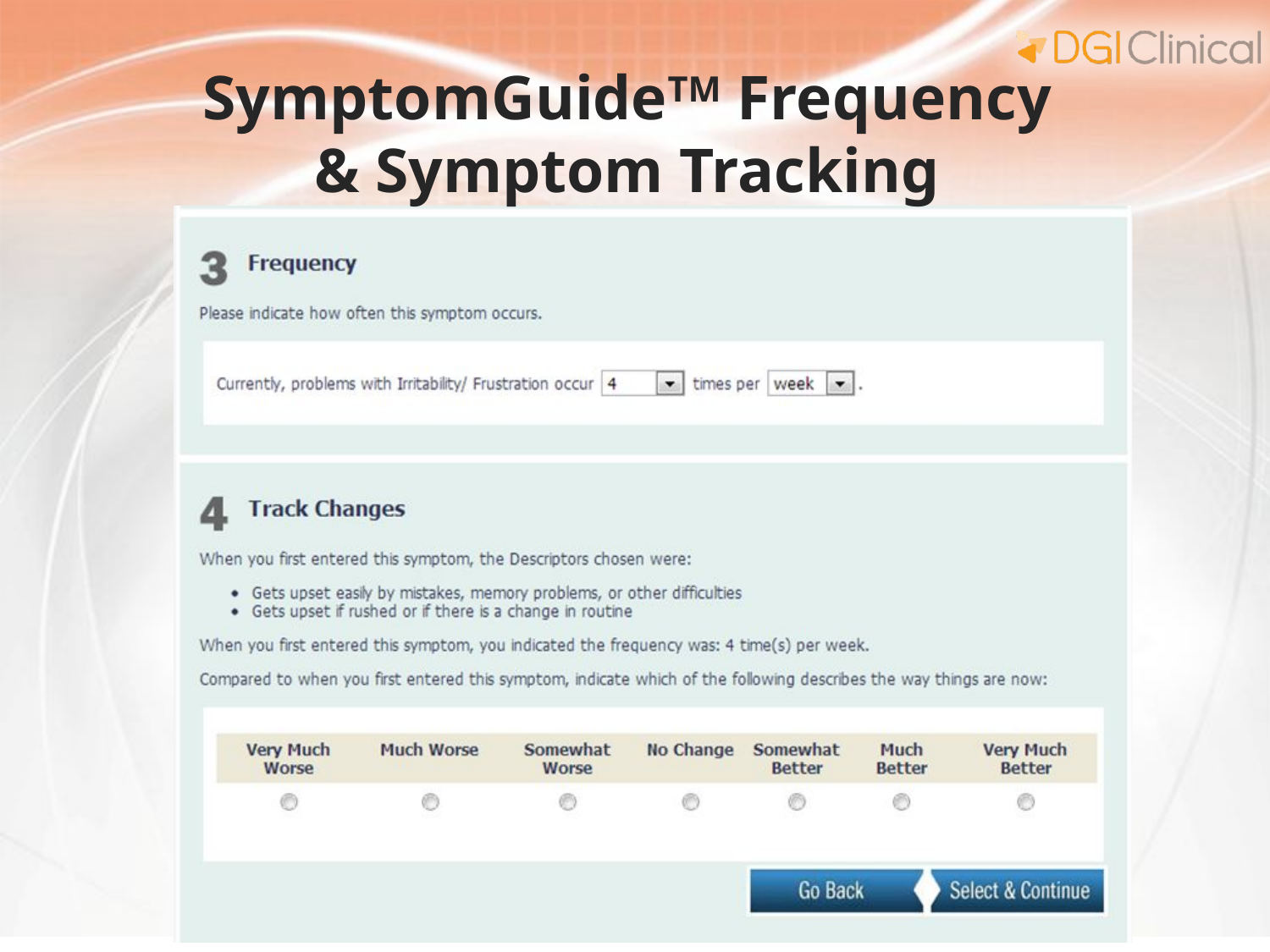

# SymptomGuideTM Frequency & Symptom Tracking
